# Health coaching for promoting physical activity in low back pain patients: a secondary analysis on the usage and acceptance

**DOI:** 10.1186/s13102-019-0154-4

**Published:** 2020-02-03

**Authors:** Lea Anna Lisa Dejonghe, Kevin Rudolf, Jennifer Becker, Gerrit Stassen, Ingo Froboese, Andrea Schaller

**Affiliations:** 1 Institute of Movement Therapy and Movement-Orientated Prevention and Rehabilitation, German Sport University Cologne, Am Sportpark Muengersdorf 6, 50933 Cologne, Germany; 2 Department of Community Health, University of Applied Sciences, Gesundheitscampus 6-8, 44801 Bochum, Germany; 3 Working Group Physical Activity-Related Prevention Research, German Sport University Cologne, Am Sportpark Muengersdorf 6, 50933 Cologne, Germany; 4 Center for Health through Sport and Movement, German Sport University, Cologne, Am Sportpark Muengersdorf 6, 50933 Cologne, Germany

**Keywords:** Usage, Acceptance, Promoting physical activity, Health coach, Face-to-face contact, Telephone coaching, Web based coaching

## Abstract

**Background:**

Multicomponent interventions combined with health coaching are widely recommended to improve a healthy lifestyle. The aim of the present study was to analyse the usage and acceptance of a multicomponent intervention (telephone, web and face-to-face coaching) for low back pain patients, and thereby gain an understanding of why this intervention was not as effective as expected.

**Methods:**

A secondary analysis of a randomised controlled trial, aimed at promoting physical activity, was conducted. It was a cross-sectional study based on data of a multicomponent intervention group (baseline = 201 participants). For evaluating the usage and acceptance, descriptive statistics were applied.

**Results:**

Over half (*n* = 118) of the patients participated at least once in the telephone coaching. Approximately half of the participants (44 of 90) rated the telephone coaching as “good”.

34 of 92 (37%) participants reported of visiting the web-platform. The web-platform was comprehensible for nearly one-quarter (*n* = 8 of 33) and very useful for one participant.

The face-to-face-contact was rated highly (range: 79.4–88.2 out of 100).

**Conclusion:**

Usage of the telephone coaching approach was moderate with even fewer participants visiting the web-platform. In addition, these approaches were not rated as very useful. The acceptance of the face-to-face contact was high.

Since the usage and acceptance could influence the effectiveness, utilisation and acceptance studies might help to explain the reason for non-effective lifestyle interventions. Therefore, more studies analysing the usage and acceptance are needed. To improve the usage and acceptance, a stronger participatory orientation in the design of interventions and the integration of face-to-face contact could be helpful.

## Background

Physical activity is recognised as a health-enhancing lifestyle factor. Therefore, the promotion of regular physical activity is important in the rehabilitative treatment of chronic disease [1]. Exercise therapy is an integral part of musculoskeletal rehabilitation in low back pain patients [2] and recommended as a successful therapy [3]. However, evidence on sustainability is lacking [4] and implementing physical activity in daily routine after rehabilitation is a common problem [5].

In behavioural interventions, various approaches for promoting physical activity are used. Most frequently, web-based, telephone-based and face-to-face contact are integrated in lifestyle interventions [6]. Currently, multicomponent lifestyle interventions, meaning interventions which combine more than one approach, are frequently recommended. These approaches are often implemented by a client-centred health coach, with the aim of motivating individuals to achieve self-determined health promoting goals [7].

However, most of the results in interventions promoting physical activity only show small to moderate effects [8, 9] and up to now, it is unknown which intervention strategy is the most effective [10, 11]. There from arises the question on why an intervention not provides the expected benefits. Lifestyle interventions are complex and their success is often dependent on various factors such as the content and implementation of it [12]. Since the usage and acceptance also could influence the effectiveness [13, 14], utilisation and acceptance studies might help to explain reasons for non-effective lifestyle interventions. It might be also promising regarding priorities in resource allocation [15]. Nevertheless, only a few studies have analysed the usage and acceptance of lifestyle interventions until now [16, 17].

The aim of the present study was to analyse the usage and acceptance of a multicomponent, health coaching intervention, which aimed to improve physical activity of low back pain patients (Movement Coaching) [18, 19], and thereby gain an understanding of why this intervention could not prove its effectiveness.

## Methods

### Study design and data source

Data was collected as part of the *Movement Coaching* research project (German Clinical Trials Register (DRKS)-ID: DRKS00004878). In brief, this randomised controlled trial evaluated the effectiveness of two interventions that promote physical activity for patients with low back pain: a multicomponent intervention (*Movement Coaching*) compared to a low level intensity intervention (control intervention) [18]. The trial had three measuring points (T0 = start of inpatient rehabilitation; T1 = six month follow-up; T2 = twelve month follow-up). Participants answered a questionnaire on sociodemographic variables, usage and acceptance, physical activity and clinical variables. The outcome data at six month (T1) and twelve months (T2) were collected using questionnaire, which was sent by letter [19].

To assess the recruitment strategy and the feasibility in the inpatient rehabilitation a pilot study was conducted.

The present secondary analysis was conducted as a cross-sectional study based on T1 data of the *Movement Coaching* intervention group. The study protocol, with the description of the intervention, has already been reported elsewhere [18].

### Study population

Participants were aged 18 to 65 years of age with a documented history of low back pain who were recruited through an inpatient centre in Germany from May 2013 to April 2014. Exclusion criteria included cognitive disorders, difficulty understanding German, surgery within the last twelve weeks, posttraumatic conditions, a current state pension claim and refusal of participating in the study.

The centre invited eligible patients to an informative meeting about the study. This meeting was held during the first week of inpatient rehabilitation. The patients had the possibility to participate in the study by giving informed consent until the first unit of the intervention [19].

### Sample description

The consort flow chart of the main study has already been published [19, 20]. At baseline (T0) 201 participants were randomised in the *Movement Coaching* intervention group.

Participant characteristics are presented in Table 1. At six months follow-up (T1), 92 (46%) participants replied to the follow-up questionnaire [19, 20].

**Table 1:** Baseline characteristics of the sample (see [19])

Age (years)	mean (SD)	49.7 (±8.3)
Gender: men	n (%)	143 (71%)
Body mass index (km/m^2^)	mean (SD)	28.9 (±5.3)
Highest level of education “lower secondary school”	n (%)	101 (50%)
Duration of low back pain > 12 months	n (%)	168 (84%)
Intensity of pain during the last four weeks (min. = 1; max. = 6)	mean (SD)	4.6 (±0.9)

*SD* standard deviation

### Intervention

All patients received three weeks of inpatient rehabilitation. In addition, the participants randomised to the intervention group were provided support by health coaches and received the theory-based multicomponent *Movement Coaching* intervention [18]. The intervention is based on the “Rubicon Model of Action Phases” [21] and the “MoVo Process Model” [22]. Additionally, contextual needs are considered within the concept of the intervention [23]. Concerning coaching methods and principles, the coach does not give any rules, concrete suggestions or solutions. The coach emphasizes the patient’s self-efficacy and individual resources to elaborate individual strategies on physical activity promotion [24].”

The intervention combined three approaches:

1. A personalised, guideline-based telephone coaching which comprised of at least two calls with the aim of providing support to the participants to integrate physical activity in daily life. If a participant could not be reached in the designated week, the coach attempted to contact the patient within the next two weeks. If a patient could not be reached within the period of three weeks, the phone coaching was deleted without replacement.
2. The opportunity to use an interactive web 2.0 online platform with further specific information on the benefits of physical activity and advice on how it helps manage low back pain, which was explained during the inpatient rehabilitation. Additionally, the patients had the opportunity to communicate with the coach or other patients on the web platform.

The participants could contact the coach via telephone or Internet whenever they wanted.

(3) Two face-to-face contact sessions of 60 min in small groups with a maximum of eight people during the inpatient rehabilitation. The main goal of these sessions was to plan the participant’s physical activity for once they had completed the inpatient rehabilitation. The face-to-face meetings were integrated in the official therapy plan of the rehabilitation centre and all patients of the *Movement Coaching* intervention group had face-to-face contact.

The health coaches were male and female with a master’s degree in the field of “prevention, rehabilitation and health management” and additional training [18–20].

Table 2 shows a summary of the three approaches; a more detailed description of the approaches has been published previously [18].

**Table 2:** Description of the movement coaching intervention (see [18])

Approach	Time	Main objectives
(1) Telephone Coaching	Week 8 after inpatient rehabilitation	Establishing a solid relationship of trust, current physical activity behaviour of the patient, barriers and facilitators to transfer physical activity plans in daily living, further planning in physical activity activities
	Week 12 after inpatient rehabilitation	Current physical activity behaviour of the patient, barriers and facilitators to transfer physical activity plans in daily living, further planning in physical activity activities
(2) Internet based aftercare	Web 2.0-platform (until 12 months after inpatient rehabilitation)	Target group specific information on physical activity and low back pain, communication platform
(3) Face-to-face contact	Inpatient rehabilitation, week 2	Motivation, perceived consequences of physical activity behaviour: Health-related risk perception, self-efficacy beliefs, planning individual physical activity after rehabilitation
	Inpatient rehabilitation, week 3	Planning individual physical activity after rehabilitation, self-efficacy beliefs, barriers and solution strategy, networking; places to be physically active at home

### Measures

Sociodemographic variables were collected at baseline.

For assessing the usage of the telephone coaching, the health coaches noted whether the participants answered the first and second call in a log. Moreover, call duration, number of call attempts until the patient was reached and reasons why the coaching did not take place were noted. For assessing the participant-related acceptance, non-standardised questions were used (see Table 3).

**Table 3:** Non-standardised questions for assessing the acceptance of the telephone coaching (translated)

Question	Operationalisation
How do you rate the telephone coaching?	6-point Likert scale (1 = very good, 6 = very bad); or “no information”
Was telephone coaching helpful in planning and executing your everyday sports or physical activities?	6-point Likert scale (1 = very, 6 = not at all); or “no information”
How much did you benefit from the call?	Not at all, somewhat, very; no information
How bothersome was the phone call?	Not at all, somewhat, very; no information

Regarding the web coaching*,* the participants were asked if they had internet access and how often they accessed the internet. Table 4 shows non-standardised questions for the subjective usage and the acceptance of the web platform. Moreover, login-data can be used to analyse the objective usage (if and how often the platform was visited).

**Table 4:** Non-standardised questions for assessing the usage and acceptance of the web-platform (translation)

Question	Operationalisation
Were you on our web-platform after inpatient rehabilitation?	Yes, No
Was the content of the web-platform easy to understand?	6-point Likert scale (1 = very easy to understand, 6 = not at all comprehensible); or “no information”
Was the web-platform helpful in planning and executing your everyday sports or physical activities?	6-point Likert scale (1 = very, 6 = not at all); or “no information”

To assess the acceptance of the face-to-face coaching, the COHEP-questionnaire (Comprehensibility of Health Education Programs) was used [25]. This questionnaire consists of four scales and comprises 30 Items (five-point Likert scale) which can be summed up to a value from 0 to 100:

1. Scale: Comprehension-fostering behaviour of program trainers = 11 Items
2. Scale: Transferability to everyday life = 9 Items
3. Scale: Comprehensibility of medical information = 6 Items
4. Scale: Amount of information = 4 Items.

Higher values indicate a higher parameter value.

### Statistical analysis

Means, standard deviations and frequencies were used for sample description and for describing the usage and acceptance. The researchers tested differences in characteristics of the repliers and non-repliers within the *Movement Coaching* intervention group using t-test and the Pearson Chi-squared test.

For all statistical tests, the significance level was set to *p* < 0.05. All analyses were run with IBM SPSS Statistics 25.

## Results

At six months follow-up (T1), 92 participants replied to the questionnaire [19, 20]. There were no statistically significant differences in the characteristics of participants who replied and did not reply within the *Movement Coaching* intervention group (*p* > 0.05).

### Telephone coaching

118 (59%) patients participated at least once in telephone coaching. 113 (56%) patients participated in the first and 99 (49%) in the second telephone coaching sessions. The most common reasons for not attending were that the participants did not provide a telephone number (*n* = 29; 14%) or that the telephone coaching was refused (*n* = 25; 12%). Duration of the calls and call attempts are shown in Table 5. 113 (telephone coaching 1) and 99 (telephone coaching 2) participants were reached, but due to missing data not for all of them data about the call duration and call attempts exist.

**Table 5:** Duration and call attempts of the first and second telephone coaching

	Telephone coaching 1	Telephone coaching 2
Call duration [min.sec] mean (SD)	07.18 (±03.04) (*n* = 111)^a^	06.53 (±03.17) (*n* = 96)^a^
Call attempts [n] mean (SD)	3.5 (±2.9) (n = 111)^a^	3.6 (±3.3) (*n* = 97)^a^

^a^ Different n due to missing data; SD standard deviation

Of the participants who answered the question “How do you rate the telephone coaching?” (*n* = 90), 49% (*n* = 44) rated the telephone coaching as very good or good (scale value 1 or 2). Ten of 84 patients (12%) benefited highly and 17% (*n* = 14) saw no benefit from the telephone coaching. 8% (*n* = 7 of 88) patients rated the telephone coaching as very useful for the planning and performance of physical activity (see Table 6).

**Table 6:** Usefulness rating of the telephone coaching

Scale	1 Very good n (%)	2 n (%)	3 n (%)	4 n (%)	5 n (%)	6 Very bad n (%)	No information n (%)
Question							
How do you rate the telephone coaching? (n = 90)	21 (23%)	23 (26%)	12 (13%)	4 (4%)	1 (1%)	1 (1%)	28 (31%)
	1 Very n (%)	2 n (%)	3 n (%)	4 n (%)	5 n (%)	6 Not at all n (%)	No information n (%)
Was telephone coaching helpful in planning and executing your everyday sports or physical activities? (*n* = 88)	7 (8%)	9 (10%)	17 (19%)	9 (10%)	9 (10%)	10 (11%)	27 (31%)
	Very n (%)		Somewhat n (%)		Not at all n (%)		No information n (%)
How much did you benefit from the call? (*n* = 84)	10 (12%)		37 (44%)		14 (17%)		23 (27%)
How bothersome was the phone call? (*n* = 85)	2 (2%)		11 (13%)		55 (65%)		17 (20%)

### Web-platform

More than three-quarters (*n* = 68; 76%) of the participants who answered the questions regarding internet (*n* = 90) have internet access and 13% (*n* = 12) do not. 11% (*n* = 10) gave no information. 52% (*n* = 47) use the internet daily and 13% (*n* = 12) never.

Thirty-four of 92 participants (37%) reported in the questionnaire that they visited the homepage at least once. Login protocols revealed that 26, of the 92 participants (28%) who answered the questionnaire, logged in on the web-platform during the intervention period. Participants who objectively used the web-platform logged in on average 1.7 (±1.1) times.

Twelve people claimed to have logged in without actually doing so. Four people stated that they did not log in, even though the platform’s log data indicated that they had logged in.

Of the 34 participants who said that they use the web-platform, 33 participants answered the question “Was the content of the web-platform easy to understand?”. 24% (*n* = 8) of those rated the web-platform as very comprehensible (scale value 1). 3% (*n* = 1) rated the web-platform as very useful (scale value 1) and 21% (*n* = 7) as not useful (scale value 5 and 6) (see Table 7).

**Table 7:** Usefulness rating of the web-platform

Scale	1 Very easy to under-stand n (%)	2 n (%)	3 n (%)	4 n (%)	5 n (%)	6 Not at all compre-hensible n (%)	No information n (%)
Question							
Was the content of the web-platform easy to understand? (*n* = 33)	8 (24%)	15 (46%)	5 (15%)	2 (6%)	0	0	3 (9%)
	1 Very n (%)	2 n (%)	3 n (%)	4 n (%)	5 n (%)	6 Not at all n (%)	No information n (%)
Was the web-platform helpful in planning and executing your everyday sports or physical activity? (n = 33)	1 (3%)	3 (9%)	16 (49%)	3 (9%)	6 (18%)	1 (3%)	3 (9%)

### Face-to-face contact

The COHEP-questionnaire was answered by 90% (*n* = 83) of the 92 patients, who answered at six month follow-up. Regarding the acceptance of the face-to-face contact, the COHEP-scales were voted with a maximum of 88.2 (±24.6) and a minimum of 79.4 (±11.5) out of 100 (scale value 2) (see Table 8).

**Table 8:** Descriptive statistics of the scales of the COHEP regarding the face-to-face contact (*n* = 92)

COHEP-Scale	n	mean (±SD)	Median [25%; 75%]-percentile
1. Comprehension-fostering behaviour of program trainers	83	86.3 (±11.6)	86 [80;94]
2. Transferability to everyday life	85	79.4 (±11.5)	80 [73;88]
3. Comprehensibility of medical information	86	85.9 (±9.7)	86 [80;93]
4. Amount of information	84	88.2 (±24.6)	75 [60;85]

SD standard deviation

## Discussion

The present study aims to evaluate the usage and acceptance of an unsuccessful multi-approach health coaching intervention for low back pain patients and to explain why this lifestyle intervention showed no effects.

More than half of the patients used the telephone coaching approach and objective login data showed that even fewer patients (28%) visited the web-platform. According to the acceptance, especially the face-to-face-contact was rated highly.

The results of this study may help explain why, in the main study of the *Movement Coaching* research project, the multicomponent approach could not prove its effectiveness compared to the control intervention results [19, 20]. The low to moderate usage might explain why it was not possible to show more beneficial effects of the intervention group.

According to our data, the telephone coaching seemed to be very demanding, even though the participants reported satisfaction, which can also be seen in other studies [26]. However, it is notable that in the current study many of the participants rated the telephone coaching as not useful for the planning and performance of physical activity. Overall, the patients rated the telephone coaching as “good”. Most participants reported that they benefitted “a little” from the telephone coaching. Other studies also show that this approach has weaknesses regarding the benefit to everyday life [27].

Interestingly, approximately a quarter of the participants reported that they had no interest in taking part in the telephone coaching, and some participants did not provide a telephone number. These results indicate that each patient has different preferences regarding an approach.

Also, a web-platform seemed to not be suitable for all patients. The present results show that approximately one quarter of the participants do not use the internet or neither had no internet access, which is similar to the average percentage of the German [28]. Nevertheless, the subjective usage of the web-platform was low, despite good ratings. Other researchers found the same: good rating and low usage [29, 30]. Often the usage of web-platforms in health interventions is low and the use time short [31]. Factors such as the design of the web-platform, frequency of updates and reminders, interactivity as well as tailored content can influence the usage [32]. However, a systematic review about “e-therapies” shows that the usage varies notably [33]. The difference between the objective and subjective usage of web-platforms is an already known phenomenon; the subjective log data is likely to be less accurate [34]. Reasons for a higher subjective usage are the dependence of the users´ memory and the social desirability [35].

An important factor, which is missing in the telephone coaching and in web-based coaching, seems to be physical interaction. Many health coaching interventions aiming to create a healthy lifestyle integrate face-to-face contact [36, 37]. Although the intervention’s effectiveness improves when the intervention integrated a combination of different approaches [38], face-to-face contact is often rated positively and seems to be very important [32, 37]. According to the COHEP-voting used in the current study, the acceptance of the face-to-face contact is high [25].

As not every approach is suitable for every patient, rehabilitation and aftercare programs need to be more individual. This is in line with Deck et al. (2015) [39], who summarized that a more flexible design of rehabilitation and aftercare is needed to improve patients’ benefit. Beside the individualization, it is important to focus on aftercare throughout the rehabilitation [40].

Limitations of the current study need to be noted. The generalisation of the results is limited, because the sample is specific. However, the intervention reached a relevant target group for physical activity promotion [19]. Being overweight and having a low education status, especially in men above the age of 30, are associated with chronic diseases [41]. Men also differ from women regarding the usage of health-promoting lifestyle interventions [42]. In general, young, highly educated women with body mass index smaller than 25 often use more approaches to enhance their health [43]. The specific sample and the rehabilitative setting should be considered in the interpretation of the results.

At six-month-follow-up, the response rate was low. Nevertheless, a pilot study was done, the response rate was not evaluated in the pilot study but taken from previous work of the research group regarding sustainability of inpatient rehabilitation. A possible reason for the high loss of participants at follow-up might be the lack of compliance with the approaches. Reasons for choosing the option “no answer” can be the non-usage but may also a lack of motivation to answer. It is notable that the number of logins provides no clear information about the intensity of the usage.

Future research should also rely on qualitative methods, as these may offer the opportunity to carry out an in-depth exploration of unanticipated and complex issues [44]. Moreover, future trials should asses all components of an intervention to make them comparable to each other. Nevertheless, another strength is the utilisation of different questions regarding the usage and the acceptance of the different approaches, and that the usage of the web-platform was analysed subjectively and objectively.

## Conclusion

To sustainably create a healthy lifestyle, in recent years, multicomponent interventions combined with health coaching are frequently recommended. As usage and acceptance may influence effectiveness of complex interventions, utilisation and acceptance studies might help to explain the reason for non-effective lifestyle interventions. Therefore, more studies should include process evaluation to understand the discrepancy between observed and expected results. Furthermore, a feasibility and piloting stage is needed [12]. To improve the usage and acceptance, a stronger participatory orientation in the design of interventions and the integration of face-to-face contact could be helpful.

The present study showed that the usage of the different approaches was generally moderate to low. However, the acceptance of face-to-face coaching in the present study was good. It is important that the patient subjectively benefit from an approach and that it is useful for the planning of the daily life.

As health coaching interventions are costly, in addition to formative process evaluations, cost-effectiveness evaluations should also be conducted.

## Data Availability

The datasets used and/or analysed during the current study are available from the corresponding author on reasonable request.

## Abbreviations

COHEP: Comprehensibility of Health Education Programs
GPAQ: Global Physical Activity Questionnaire
MoVo Process Model: Motivations-Volitions-Prozessmodell
SD: Standard deviation
